# Analytical method development and validation for the quantification of metaldehyde in soil

**DOI:** 10.1016/j.mex.2021.101482

**Published:** 2021-08-08

**Authors:** Nathan Keighley, Carmel Ramwell, David Werner, Chris Sinclair

**Affiliations:** a Fera Science Ltd. York Biotech Campus, Sand Hutton, York YO41 1LZ, UK; b School of Engineering, Newcastle University, Newcastle upon Tyne NE1 7RU, UK

**Keywords:** Soil extraction, Matrix suppression, Optimized recoveries

## Abstract

Previously published methods for the analysis of metaldehyde were adapted for its reliable quantification in soil extracts. Varied methanol-water extraction solvents were trialed, but the use of pure methanol proved to be the most reliable approach for the scaled down methodology. Analysis of metaldehyde was done using LC-MS. Initially the method had problems with matrix suppression of the signal. The method was therefore further developed to overcome this challenge to avoid the costs and time demands of laborious clean-up protocols. This modification to the method involved use of the BEH Phenyl column instead of the C_18_ column initially used, and optimization of the gradient flow of the mobile phase. The optimized LC-MS method was validated and used for further research applications. In brief,

• We investigated the recovery of metaldehyde from spiked soil samples.
• The optimized LC-MS method achieved acceptable metaldehyde recoveries (100–132%, 109% on average) for a range of soil types.
• The optimized method was suitable for high through-put analyzes.

## Graphical abstract

**Figure:**
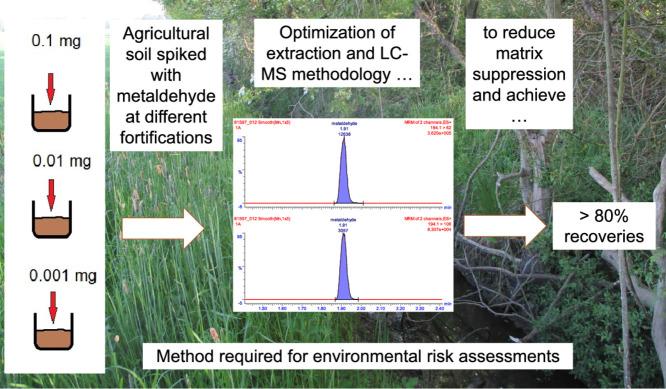

Specifications table

**Table:** 

Subject area	Environmental science
More specific subject area	*Environmental fate assessments*
Method name	Extraction and LC-MS analysis of metaldehyde in soil samples
Name and reference of original method	Extraction methodology adapted from EFSA [1]; LC-MS analysis developed from Thomas et al. [2]
Resource availability	*N.A.*

## Analytical challenges

Quantifying metaldehyde, the active ingredient in molluscicide, in soil extracts by LC-MS presented a challenge for the concentration range anticipated in biodegradation batch studies. Challenges include quantitative extraction from different soil types, and interference of matrix components from the extracts with the LC-MS analysis of the target compound metaldehyde, especially at low concentration. Several extraction procedures were trialed, and techniques were adopted to mitigate matrix effects in the LC-MS analysis. These included the use of matrix-matched analytical standards, dilution of the methanol extracts with water, and finally an optimized LC-MS procedure for the improved separation of metaldehyde from interfering compounds using a BEH phenyl column (Acquity UPLC).

## Soil types, metaldehyde spiking and extraction procedures

Methods in the literature [1] that detail metaldehyde extraction from soil tend to use methanol, or methanol/water solvents. This study based the extraction method on the published methods [1]. Initial method development to extract metaldehyde used a pasture clay loam soil, which was sampled from a livestock field in North Yorkshire and free from any known previous metaldehyde application. Soil samples (50 g dry weight basis) were weighed into 250 ml plastic screw-top containers, the moisture adjusted to 60% of maximum water holding capacity (MWHC) using deionized water and then left to settle for 30 min. The soil samples were spiked with metaldehyde (Sigma Aldrich, 99% purity) dissolved in water at three fortification levels (2.0, 0.2, and 0.02 mg kg soil^−1^) and left to equilibrate for 30 min. Extraction of metaldehyde comprised the addition of methanol (100 ml, analytical grade from Sigma Aldrich), shaking the containers at 200 rpm for 30 min on a side-to-side shaker, followed by centrifuging (Beckman Coulter, Allegra) for 5 min at 3500 rpm and decanting the supernatant into a centrifuge tube. This process was repeated in a second extraction using methanol (50 ml) and the second extract was stored separately. The samples were stored in a refrigerator at 4 °C prior to analysis by liquid chromatography mass spectrometry (LC-MS). Three replicates were used as well as an untreated control.

In the second stage of method development, a range of standard soils (Lufa-Speyer), covering different texture classes and organic matter contents (refer to Table 1), were selected to validate the extraction method for a range of soil types. The soil properties were provided by Lufa-Speyer with the exception of the pasture clay loam (CL), whose properties were measured by Forest Research UK. The validation aimed to demonstrate that metaldehyde can be extracted with high recovery (70–110%) from a range of soils with different clay contents, organic matter contents and pH values. Soil samples (20 g dry weight basis) were weighed into centrifuge tubes and moisture adjusted to 60% MWHC with deionized water. Three replicates were used as well as an untreated control. The samples were spiked with metaldehyde at two fortification levels (1.5 and 0.15 mg kg soil^−1^), thoroughly mixed with a spatula, and extracted with 25 ml methanol on a side-to-side shaker (200 rpm for 30 min), centrifuged (5 min at 3500 rpm) and the supernatant poured off and stored in a vial (Extract A). This process was repeated for a second sequential extraction (extract B). The extracts A and B were stored in separate vials in a refrigerator at 4 °C. The fortification concentrations of 1.5 mg kg soil^−1^ and 0.15 mg kg soil^−1^ were chosen as being approximately the same as the fortification concentration typically used in batch experiments, then a tenth of that value, respectively. Further validation samples were produced at a one-hundredth dose (0.015 mg kg^−1^), to test the limits of detection.

**Table 1:** pH, organic carbon (OC) content and texture of the validation soils.

Soil sample	pH (CaCl_2_)	OC%	Sand%	Silt%	Clay%
**Pasture CL**	6.3	2.73	43.0	29.0	28.0
**2.1 soil**	4.7	0.67	86.1	10.2	3.7
**2.3 soil**	5.9	0.66	59.1	33.3	7.6
**2.4 soil**	7.4	1.99	32.1	41.3	26.3
**6S soil**	7.2	1.78	23.8	35.3	40.9

CL = clay loam

## Method optimization

Initially, complications arose when metaldehyde extractions were performed at low spiking concentrations of 10 and 1% of the fortification level, representative of expected concentrations to occur at 90 and 99% degradation. At low concentration, recovery of metaldehyde was very poor (< 10%). These results are shown in Table 2. A concentration-dependent process was apparently suppressing the metaldehyde signal during LC-MS analysis, and further experimental work was undertaken to understand the phenomenon.

**Table 2:** Recoveries (% quantified relative to application) of metaldehyde for the validation of extraction at different concentrations in the pasture clay loam soil, not accounting for matrix suppression.

Fortification level (mg/kg)	Extraction	Mean recovery (% application)					Mean	RSD
		Rep 1	Rep 2	Rep 3	Rep 4	Rep 5		
**0.015**	A	0	0	0	0	0	0	n/a
	B	0	0	0	0	0	0	n/a
**0.15**	A	3.79	4.41	1.22	1.38	0	2.70	1.42
	B	0	0	0	0	0	0	n/a
**1.5**	A	54.4	53.4	52.0	-	-	53.27	0.98
	B	22.9	21.9	-	-	-	22.43	0.41

The extraction method was trialed with other solvent systems, namely water alone and acetonitrile, concurrently to see if other solvents would perform better, but again, recoveries were equally poor (< 10%). It was hypothesized that the observed poor recovery could be due to irreversible sorption to the soil, or matrix effects influencing the LC-MS analysis. Experiments were designed to test these ideas. Further extractions were done on a sandy soil, with low organic carbon content, compared to the organic-rich clay loam initially used in the study, with the expectation that sorption would be less relevant for the sandy soil. In this experiment also, recovery was poor for both soils, indicating that sorption of metaldehyde was not the main cause for poor recoveries. Direct spiking of blank extracts (50 ml) with 0.01 mg of metaldehyde was done to avoid sorption to soil. Despite the expected concentration being double, and no soil available to cause sorption, recovery was still below 20%. It was concluded that matrix effects were influencing the LC-MS analysis.

**Fig. 1:**
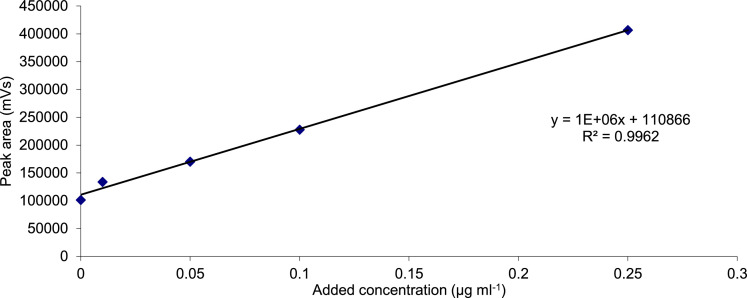
Standard addition plot (external standardization) for metaldehyde standards made up in the supernatant of a 0.01 mg fortification in 100 ml supernatant extraction from soil.

**Fig. 2:**
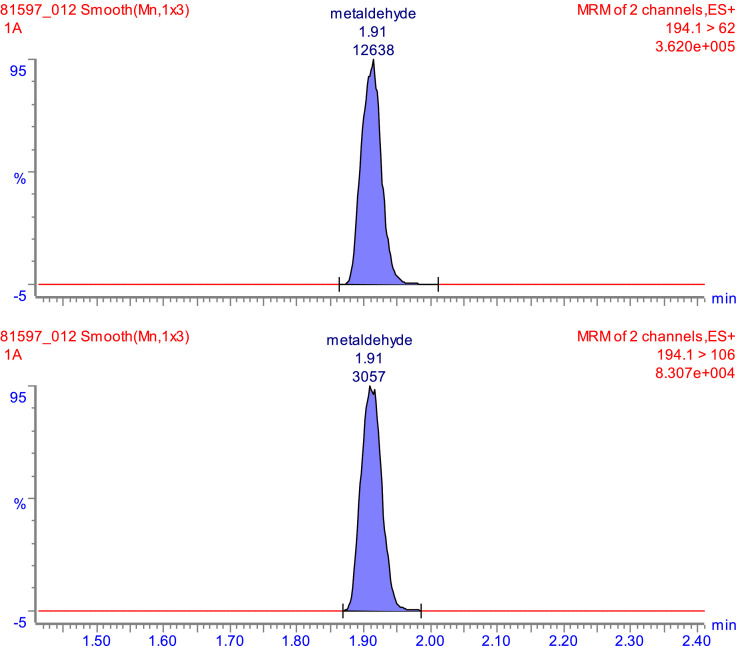
Chromatograms from the optimized LC-MS method showing both transitions at m/z values of 62 and 106 for a matrix-matched calibration standard of 0.25 µml^−1^ metaldehyde in methanol.

To better understand how matrix suppression might be influencing the LC-MS results at lower concentration in the supernatant, standard addition samples were prepared and run on the LC-MS instrument. The standard addition samples were made up from metaldehyde stock solution (0.01 mg/ml in methanol) then diluted in 10 ml volumetric flasks using the supernatant from extraction of 0.01 mg fortification of metaldehyde in 50 g soil with 100 mL methanol. This was done in accordance with the aforementioned extraction method, using the supernatant from the first extraction. By diluting different quantities of the stock solution, standards of 0.01, 0.05, 0.1 and 0.25 µg ml^−1^ were prepared, not accounting for the metaldehyde present in the supernatant, along with a sample of the supernatant alone. A standard addition plot (Fig. 1) was produced of the peak area (from the chromatograms, such as the one in Fig. 2) against the concentrations of the standards and supernatant alone to give a linear plot from which the concentration of metaldehyde in the supernatant could be calculated without influence from matrix suppression. This technique yielded a recovery of 110.9% for the extraction of 0.01 mg metaldehyde from 50 g soil, which was vastly improved from previous attempts.

However, producing standard additions greatly increases the number of samples to be measured. Given that matrix suppression had been confirmed to be an issue in the analysis of metaldehyde, work on the LC-MS method was undertaken for optimization against matrix effects. Different LC columns were trialed (Kinetex XB-C18 50 × 2.1 mm column (Phenomenex, Macclesfield, UK and BEH phenyl (Acquity UPLC)), and alterations to the LC-MS method adapted from Thomas et al. [2] were made to optimize the analysis of soil extracts, rather than extracts from bacterial cultures, as was the case in the referenced study.

**Fig. 3:**
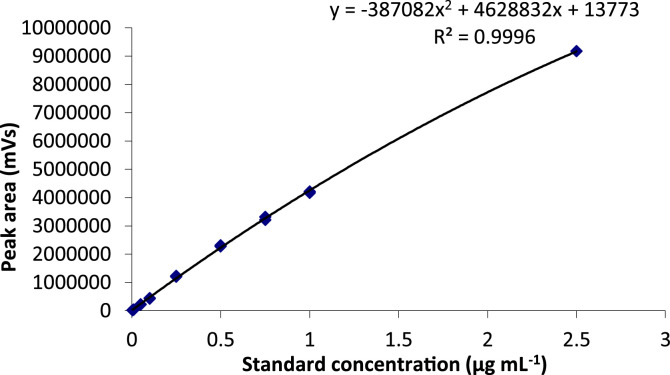
Calibration curve used for the method validation developed for the extraction of metaldehyde from soil.

Samples for LC-MS analysis were prepared by diluting 1/10 with water and filtered through a (*Whatman*) 0.2 μm pore nylon filter (*Thermo Fischer Scientific*). Calibration standards were matrix matched, produced from the same metaldehyde stock solution used for spiking the batch experiments, spiked into blank soil extract. The calibration range used included the following standards: 0.01 µg ml^−1^, 0.05 µg ml^−1^, 0.1 µg ml^−1^, 0.25 µg ml^−1^, 0.5 µg ml^−1^, 0.75 µg ml^−1^, 1.0 µg ml^−1^, and 2.5 µg ml^−1^. Calibration standards were produced by serial dilution of the 0.1 mg ml^−1^ metaldehyde (aq) stock solution used for the soil spiking. These were matrix-matched by amending the standards to 1/10 methanol used in a blank extraction of the test soil. A calibration curve is shown in Fig. 3.

The LC-MS instrument used initially, during the analysis of these samples, was a Shimadzu Nexera X2-SCIEX Qtrap 5500. The LC-MS method was developed from the one described by Thomas (2016) and optimized for metaldehyde in soil extracts. A Kinetex XB-C18 50 × 2.1 mm column (Phenomenex, Macclesfield, UK) was used with 2.6 μm particles, with a flow rate of 0.5 ml min^−1^, using a mixture of 1 mM ammonium acetate, prepared with ultrapure water, and methanol as the organic component, and these two solvents comprised the mobile phase with an elution gradient flow detailed in Table 4. Samples were stored in a refrigerator at 4 ⁰C prior to injection into the instrument. Mass spectrometry involved an optimized protocol with greater response and improved precision that used 0.5 ml min^−1^ flow rate, 5.25 kV capillary voltage and 325 °C solvation temperature. To observe product ions (transition at m/z 106, C_4_H_8_O_2_ + NH_4_^+^, and m/z 62, C_2_H_4_O + NH_4_^+^), multiple reaction monitoring with a dwell time of 160 ms was used.

For the final method, a different LC-MS instrument (Waters Acquity-Quattro Premier XE) was used with an optimized methodology for detecting metaldehyde in soil extracts. The methodology was scaled down from the previous trial to save laboratory consumables. To avoid the issues with matrix suppression, the column was changed during the development of the optimized method. Changing the C18 column used on the SCIEX instruments to a BEH phenyl column on the Waters instruments helped to notably reduce matrix suppression of the metaldehyde signal, which caused a loss of response at low metaldehyde concentrations. The method used a BEH phenyl (Acquity UPLC) 1.7 µm particles 2.1 × 100 mm column, with a flow rate of 0.4 ml min^−1^, using the same mobile phase as previously described: 1 mM ammonium acetate, prepared with ultrapure water, and methanol as the organic component, with the same gradient flow (Table 4). For mass spectrometry, the original parameters used with the SCIEX instruments were selected, with the following amendment: opened the acquisition window earlier (between 0.5–5.00 min) with a 0.6 s solvent delay. This LC-MS method proved successful in overcoming the issues presented by matrix suppression of the metaldehyde signal, and the complete analytical method was then validated for use in environmental fate studies in metaldehyde.

## Method validation

Method validation results are presented in Table 3. The recovery of applied versus quantified metaldehyde was 100–132% for all soil types, 109% on average, with acceptably low relative standard deviation of < 20%. The proportion of metaldehyde present in the second sequential extraction (extract B) was higher for the more clayey, high organic content soil types, soil 2.4 and soil 6 S, illustrating the need for the second extraction step. These results demonstrated that applied metaldehyde could be extracted and quantified with a high recovery from different soil types.

**Table 3:** Method recoveries for the extraction of metaldehyde from different soil types (specifications in Table 1). RSD = relative standard deviation.

Soil	Mean recovery (*n* = 3) for 1.5 mg kg^−1^ fortification			Mean recovery (*n* = 3) for 0.15 mg kg^−1^ fortification		
	Extract A%	Extract B%	RSD for combined extracts	Extract A%	Extract B%	RSD for combined extracts
**2.1 soil**	82.6	27.7	9.52	113.3	0	8.54
**2.3 soil**	76.5	23.9	12.08	106.5	0	20.6
**2.4 soil**	85.6	17.4	8.33	52.2	52.5	12.18
**6S soil**	86.3	19.1	10.19	41.4	90.8	13.34

**Table 4:** Gradient flow used in the LC-MS methodology to elute metaldehyde.

Time (min)	% Ammonium acetate	% Methanol
Initial	95.0	5.0
1	50.0	50.0
3	10.0	90.0
7	10.0	90.0
7.10	95.0	5.0
12.00	95.0	5.0

## Declaration of Competing Interest

The authors declare that they have no known competing financial interests or personal relationships that could have appeared to influence the work reported in this paper.
